# Supplementary material for: Effect of the 16S rRNA Gene Hypervariable Region on the Microbiome Taxonomic Profile and Diversity in the Endangered Fish Totoaba macdonaldi
Source: Microorganisms. 2024 Oct 23;12(11):2119. doi: 10.3390/microorganisms12112119 (PMC11596169; doi:10.3390/microorganisms12112119)
Supplement: Supplementary file 1 [file microorganisms-12-02119-s001.zip › microorganisms-3164074-supplementary.pdf]

Supplementary Table

Table S1. Regions of the 16S rRNA gene and primer sequences utilized for their amplification.

Region	Sequence F (5´-3´)	Sequence R (5´-3´)	Amplicon Size
V1-V2	AGMGTTYGATYMTGGCTCAG	GCTGCCTCCCGTAGGAGT	≈ 310 pb
V2-V3	AGTGGCGGACGGGTGAGTAA	CCGCGGCTGCTGGCAC	≈ 430 pb
V3-V4	CCTACGGGNGGCWGCAG	GACTACHVGGGTATCTAATCC	≈ 450 pb
V5-V7	AACMGGATTAGATACCCKG	ACGTCATCCCCACCTTCC	≈ 394 pb

Table S2. PCR conditions for the primers used to amplify each region.

		**V1-V2**	**V2-V3**	**V3-V4**	**V5-V7**
**PCR conditions**	Initial denaturation	98 °C × 30 s	95°C for 3 min	95°C for 3 min	94◦C for 3min
	Denaturation	35 cycles at 98 °C × 10 s	35 cycles at 95°C x 30 s	35 cycles at 95°C x 30 s	35 cycles at 94 ˚C x 1min
	Annealing	60 °C × 30 s	60 °C × 30 s	55°C x 30 s	53 ˚C x 1min
	Extension	72 °C × 30 s	72°C x 30 s	72°C x 30 s	72 ˚C x 1min
	Plus final extension	72 °C × 5 min	72°C x 5 min	72°C x 5 min	72 ˚C x 10 min
**Primers name**		27F-338R	BV2F-BV3R	IlluminaF-IlluminaR	799F-1193R
**Reference**		Walker et al., 2020	Bukin et al., 2019	Klindworth et al., 2013	Beckers et al.,2016
